# CREATIVE QUALITATIVE RESEARCH METHODS WITH LESBIAN, GAY, BISEXUAL, TRANSGENDER, AND QUEER (LGBTQ+) OLDER ADULTS

**DOI:** 10.1093/geroni/igac059.539

**Published:** 2022-12-20

**Authors:** Austin Oswald, Sara Bybee, Austin Oswald

## Abstract

The voices of lesbian, gay, bisexual, transgender, and queer (LGBTQ+) older adults are very often overlooked in research contexts. Creative qualitative methods have been utilized to study populations who have been neglected, empowering marginalized communities, and fostering equitable research processes and outcomes (Archibald & Blines, 2021; Jen & Paceley, 2021; McGarry & Bowden, 2017). This innovative symposium explores creative qualitative methods of data collection and analysis, such as creative writing and poetry, which have been employed in research about LGBTQ+ aging and also describes how each method may provide a unique contribution to the research process and literature. The first presentation describes the process of facilitating a weekly creative writing group with LGBTQ+ older adults and how creative writing can facilitate the retelling of life events and reimaging of new futures. The second presentation describes the process of analyzing pieces of creative writing in order to elucidate the potential and possibility of queer futurities and their implications for research on aging trajectories and imaginings. The third presentation details how found poetry created from dyadic semi-structured interviews sheds new light on the relationships of LGBTQ+ couples facing cancer. Through these three presentations, we will illustrate how creative methods contribute strengths of generating evocative and poignant narratives, illuminating not-yet-possible futures, and inspiring equally creative interventions. The overall objective of this symposium is to explore creative qualitative research methods for their utility in research with LGBTQ+ older adults, ultimately fostering more inclusive and nuanced research processes and products.

